# Resection of multifocal intrahepatic cholangiocarcinoma

**DOI:** 10.1093/bjs/znaf048

**Published:** 2025-04-01

**Authors:** Kjetil Søreide

**Affiliations:** Department of Gastrointestinal Surgery, Stavanger University Hospital, Stavanger, Norway; Department of Clinical Medicine, University of Bergen, Bergen, Norway

Intrahepatic cholangiocarcinoma is not among the most frequent of gastrointestinal cancers. However, it is the second most common primary liver cancer (after hepatocellular carcinoma) and—for not entirely clear reasons—the incidence and mortality rate related to intrahepatic cholangiocarcinoma is increasing^1^. In contrast to hepatocellular carcinoma, which develops on the background of chronic liver disease where development of multiple lesions may represent several primary tumours due to the widespread field effect, most patients with intrahepatic cholangiocarcinoma do not have underlying exposure to any chemicals, no known primary sclerosing cholangitis, or any known cirrhosis, and usually have a normal liver. Hence, multifocal disease in intrahepatic cholangiocarcinoma may represent metastatic spread rather than multiple unique tumours^2^. Unfortunately, most patients with intrahepatic cholangiocarcinoma are not amenable to surgical treatment at time of diagnosis due to disseminated or advanced disease. However, resection is the basis for treatment attempted at cure. The consensus is to resect is fairly straightforward for smaller, solitary lesions based on recent guidelines^1,3,4^, but beyond these criteria the landscape becomes muddy. The decision to resect, the use of imaging modalities and the overall treatment in intrahepatic cholangiocarcinoma is variable between centres^5^. In the past, systemic treatment options have been few and not very effective. It is not until more recently that drugs targeting directly the underlying mutational profile^6^ or the underlying immune-dependent mechanisms^7^ have become available, with potential druggable targets and molecular prognostic information to guide treatment^8^. Going forward, this may change the perspective of tumour behaviour and cancer biology in intrahepatic cholangiocarcinoma.

**Fig. 1:**
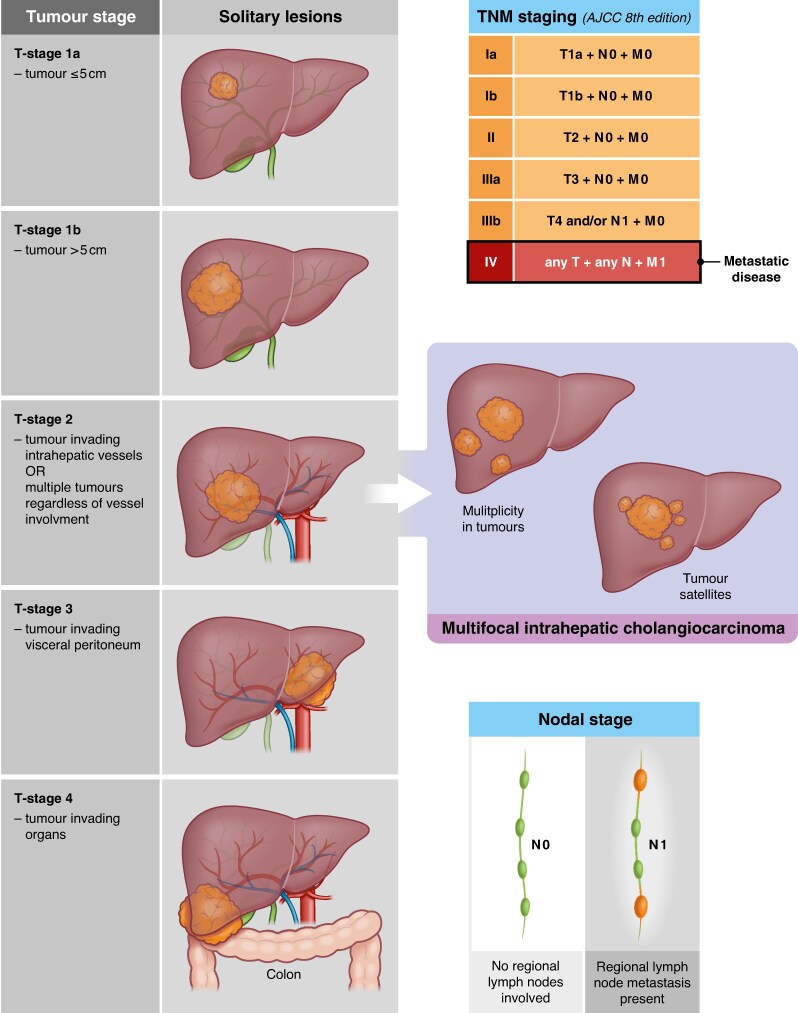
Staging for intrahepatic cholangiocarcinoma

Resection for intrahepatic cholangiocarcinoma has been entertained in patients with a good performance status, who have no extrahepatic disease, and for whom a planned radical resection (R0) with a sufficient future liver remnant could be done. However, for patients with multiple tumours (*Fig. 1*) the role of resection has been controversial. Multifocal intrahepatic cholangiocarcinoma, either as tumour satellites or several lesions within the same liver, has been regarded as a prognostically very poor sign and in many centres considered a contraindication to surgery. Some have even proposed a new staging system, where intrahepatic lesions (as metastasis) are defined as M1a disease^2^ due to the very poor prognosis, and hence not recommended for surgery. A comprehensive meta-analysis found that both the presence of either satellites outside the primary tumour or any multiplicity of tumours were strong negative prognostic factors^9^. The exact indication for surgical resection in recent guidelines is rather vague, but the European Association for Study of the Liver guidelines suggest that multifocal yet unilobar disease can be considered for resection in select patients^1^. The British guidelines simply state that ‘a complete (R0) resection with an adequate liver remnant is the preferred surgical treatment’^4^, without discussing any further contraindication or poor prognostic determinants for not proceeding to surgery. The European Society for Medical Oncology guidelines do not elaborate much on the role of surgery for intrahepatic cholangiocarcinoma^3^. Hence, huge variation in clinical practice is perhaps explained best by few leads to guide best practice of care. We need more and better data.

Multifocal intrahepatic cholangiocarcinoma represents a controversy in staging and resectability evaluation. Some have proposed multifocality to be declared as metastatic disease, and labelled as ‘M1a’ (intrahepatic metastasis) with markedly reported worse prognosis even for N0 disease

In this issue of the BJS^10^, the investigators of the International Intrahepatic Cholangiocarcinoma Study Group have queried their database for all patients with multifocal intrahepatic cholangiocarcinoma. From about 1500 eligible patients, some 208 had multifocal tumours (about 14%) of which among these about two-thirds had satellite tumours and one-third had intrahepatic metastasis. The median number of lesions was 3 and median size 7 cm. Of note, 41 (19%) patients had bilateral multifocal disease. Unsurprisingly, patients with multifocal intrahepatic cholangiocarcinoma had a high rate of major hepatectomy (about 70%) and were more likely to undergo lymphadenectomy compared to patients with solitary lesions. Multifocal tumours were more often associated with node-positive disease and several other negative prognostic factors, including morphological subtypes of invasive growth, that is the periductal infiltrating/mass-forming plus periductal infiltrating type. Perineural invasion was not prognostic in uni- or multivariable analyses, despite this being reported as a strong negative predictor in a previous cohort by the same investigators^11^. One may speculate if this negative factor is not prognostic in the setting of multifocal disease, reflecting an already aggressive biology.

Using the tumour burden score (TBS), consisting of a combination of size and numbers, the investigators found that patients with a low TBS (defined by a cut-off <7.0) who had multifocal tumours experienced a 3-year overall survival comparable to individuals with stage II/III solitary tumour (43.4% *versus* 43.2% survival rate respectively). However, the multifocal disease with low TBS represents a limited group, with only 75 patients fitting the category. Of note, more than half were no longer alive at the 12-month mark.

The current study must be viewed in light of several considerations. Despite being a large study on a disease for which few patients are resected, one has to envision that a preselected cohort exists even when resection proceeded at a time during which multifocal disease has been considered as a rather poor prognostic sign. Hence, one must assume that the cohort represents select outliers in a preoperative decision process. One must then consider how representative the selected group of resected, multifocal intrahepatic cholangiocarcinomas are to all patients with multifocal disease. About one-third of patients had undergone preoperative PET scan, a modality now recommended as routine before resection in guidelines^1,4^ to rule out extrahepatic, node positive, or distant disease. Furthermore, even in the absence of solid evidence, the clinical practice is increasingly reported towards giving neoadjuvant therapy in patients who present with findings of prognostically poor biology, such as size, number, and proximity to vessels^12^. In the current study in BJS^10^, all of these were treatment-naïve and none of the patients had received a neoadjuvant treatment for downstaging purposes, for conversion from unresectable state or to simply test for time and biology. Today, a higher number of centres may chose a trial of preoperative treatment (neoadjuvant or downstaging)^12^, even with the lack of hard data to support this. Also, guidelines now recommend biopsy for tissue diagnosis and broad mutational analysis screening at time of diagnosis^1,3,4^, as there is increasingly a focus on targeted therapies and druggable mutations for this group that may render patients eligible for specific drugs and—hopefully—better response rates. The tumour burden score suggested for selection of multifocal disease does identify a select group of patients who could be resected with favourable outcomes in a dismal disease. Eventually then, when adjusted with the addition of other molecular features that renders better systemic treatment, surgery may not only be feasible but turned into effective therapy for durable results.
